# Frailty Level Classification of the Community Elderly Using Microsoft Kinect-Based Skeleton Pose: A Machine Learning Approach

**DOI:** 10.3390/s21124017

**Published:** 2021-06-10

**Authors:** Ghasem Akbari, Mohammad Nikkhoo, Lizhen Wang, Carl P. C. Chen, Der-Sheng Han, Yang-Hua Lin, Hung-Bin Chen, Chih-Hsiu Cheng

**Affiliations:** 1 Department of Mechanical Engineering, Qazvin Branch, Islamic Azad University, Qazvin 341851416, Iran; g.akbari@aut.ac.ir; 2 Department of Biomedical Engineering, Science and Research Branch, Islamic Azad University, Tehran 1477893855, Iran; m.nikkhoo@srbiau.ac.ir; 3 Bone and Joint Research Center, Chang Gung Memorial Hospital, Taoyuan 33333, Taiwan; 4 School of Biological Science and Medical Engineering, Beihang University, Beijing 100191, China; lizhenwang@buaa.edu.cn; 5 Department of Physical Medicine & Rehabilitation, Chang Gung Memorial Hospital at Linkou and College of Medicine, Chang Gung University, Taoyuan 33302, Taiwan; chendc@adm.cgmh.org.tw; 6 Department of Physical Medicine and Rehabilitation, Bei-Hu Branch, National Taiwan University Hospital, Taipei 10845, Taiwan; dshan@ntu.edu.tw; 7 School of Physical Therapy and Graduate Institute of Rehabilitation Science, College of Medicine, Chang Gung University, Taoyuan 33302, Taiwan; linyh@mail.cgu.edu.tw (Y.-H.L.); d95548011@ntu.edu.tw (H.-B.C.)

**Keywords:** frailty level, Kinect data, machine learning, feature extraction, classification

## Abstract

Frailty is one of the most important geriatric syndromes, which can be associated with increased risk for incident disability and hospitalization. Developing a real-time classification model of elderly frailty level could be beneficial for designing a clinical predictive assessment tool. Hence, the objective of this study was to predict the elderly frailty level utilizing the machine learning approach on skeleton data acquired from a Kinect sensor. Seven hundred and eighty-seven community elderly were recruited in this study. The Kinect data were acquired from the elderly performing different functional assessment exercises including: (1) 30-s arm curl; (2) 30-s chair sit-to-stand; (3) 2-min step; and (4) gait analysis tests. The proposed methodology was successfully validated by gender classification with accuracies up to 84 percent. Regarding frailty level evaluation and prediction, the results indicated that support vector classifier (SVC) and multi-layer perceptron (MLP) are the most successful estimators in prediction of the Fried’s frailty level with median accuracies up to 97.5 percent. The high level of accuracy achieved with the proposed methodology indicates that ML modeling can identify the risk of frailty in elderly individuals based on evaluating the real-time skeletal movements using the Kinect sensor.

## 1. Introduction

Frailty is a common geriatric syndrome of physiological decline, with associated weakness, slowness, low activity, etc., which can be described as a clinical state of increased vulnerability to unfavorable health outcomes [1,2]. It can be associated with increased risk for incident disability, falls, hospitalization, and mortality [2]. Early detection of frailty symptoms can provide timely interventions and physical treatments, which may reduce its undesirable impact [3,4,5]. The Fried’s frailty phenotype [2] has been widely utilized for patient classification in most of relevant published studies in the literature [6]. This technique was based on evaluation of the criteria representing compromised energetics, including unintended weight loss, exhaustion, slowed walking speed, low griping strength, and low physical activity [2]. However, a foremost obstacle to achieve early detection of elderly frailty has been the lack of a gold standard for diagnosis and adopting the effective target treatments [2,7,8]. On the other hand, most of the classification tools to evaluate the frailty levels are more appropriate for research investigations [6,7] and it could be beneficial to enhance them for clinical applications.

Frailty can be identified based on evaluation of the impairment in daily physical activities [9] and numerous studies utilized different techniques and approaches for this purpose. The wearable sensors [10], including accelerometers [11], radio frequency detectors [12], gyroscopes [13], and inertial measurement unit-based mobile devices [14], were frequently used to assess the patient’s impairment and disability [15]. While the wearable sensors are light and can be easily used in research laboratories, the quality of collected signals depends on correct positioning to reduce the possible noise. Optical motion capture cameras were commonly used as an accepted method of patient’s motion analysis [16,17]. This technique is intrusive, expensive and not robust enough for clinical applications and remote diagnosis. A Microsoft Kinect sensor was introduced as a low cost, robust, marker free, and reliable device which could be capable of recording proximity and depth data in real time [18,19,20,21]. The Kinect could be used as a promising technology for providing reliable solutions for patient movement analysis in clinics [19,22]. Nonetheless, the reliability and validity of this technique depends on the interpretation of registered data, which highlights the importance of developing the appropriate clinical predictive models.

Machine learning (ML) modeling was recently applied in numerous studies to enhance the analysis of human motion [23] which can be beneficial for evaluation of the patient’s impairment [24,25] and prediction of fall risk and assessment of stability. ML methodology has been employed in different studies to recognize human gait datasets recorded with the Kinect sensor [23,26]. Implementation of a well-designed sequence of data process was frequently recommended as a mandatory requirement in order to convert the raw Kinect data into effective input features for the ML estimators and consequently enhance their accuracy in classification of subject’s gender [23,27]. Extraction of the anthropometric and gait features from the Kinect data and applying suitable methods for cycle identification and data quantization have been some of commonly used procedures in this regards [28,29,30]. Besides, ML was also used to identify the aging effects on Timed-up-and-Go (TUG) tests [31,32]. Other studies applied this technique to detect falls by analyzing the produced sound [33], and the possibility of using the fall detection algorithms on mobile phones were approved [34]. Alternatively, some studies were developed to use a comprehensive administrative health database of clinical characteristics and socioeconomic factors to classify the frailty level and predict the frailty condition using ML approaches [35,36], or to define the multi-morbidity frailty index by selecting disease items and ranking their importance accordingly [37]. The above mentioned studies applied the ML technique to detect specified frailty-related factors or to categorize the frailty level using vast health database. Given the fact that conventional frailty measurement methods, such as Fried’s frailty phenotype, routinely requires trained specialist and equipment chosen across a range of physiological and performance domains, the ML technique is potential to promote the self-health management and to reduce the manpower cost of health care.

Providing a real-time ML classification tool to consider muscle strength, aerobic endurance, and mobility, which can be represented as the postural transitions and execution of different motor tasks, could be beneficial for early identification of the risk of frailty. This methodology can be introduced as a robust assessment tool to enhance the accessibility of the frailty classification. Hence, the main contribution of this study is to assess the feasibility of frailty prediction using ML methodology based on the comprehensive dataset composed of features extracted from the Kinect sensor.

## 2. Materials and Methods

### 2.1. Data Acquisition and Description

#### 2.1.1. Subjects

Seven hundred and eighty-seven community elderly (aged 54–90 years, 514 females and 273 males) participated in this study. Individuals were excluded from this study if they had (1) severe cardiopulmonary disease who could not maintain activity for more than 10 min, (2) severe visual or hearing impairment to have trouble getting information on the screen, and (3) disability to manipulate with both hands. Signed informed consents were acquired from all participants prior to their enrolment in this clinical protocol, which was approved by the institutional medical research ethics committee. Adequate explanations about the experimental test procedure were given to all participants prior to commencement of the experiments.

#### 2.1.2. Experimental Test Procedure

Subjects were originally classified into the healthy (N=323), pre-frailty (N=444), and frailty groups (N=20) by the Fried’s frailty score (FS) [2], which was calculated as follows:

(1) FS=WL+WK+PE+SL+LA

where *WL*, *WK*, *PE*, *SL*, and *LA* represents the weight loss, weakness, poor endurance, slowness, and low physical activity, respectively. All of these characteristic terms are binary metrics which were defined based on the Fried’s guideline (Table 1) [2]. The criteria for a subject to be identified as ‘healthy’, ‘pre-frail’ or ‘frail’ are also listed in Table 1.

**Figure 1:**
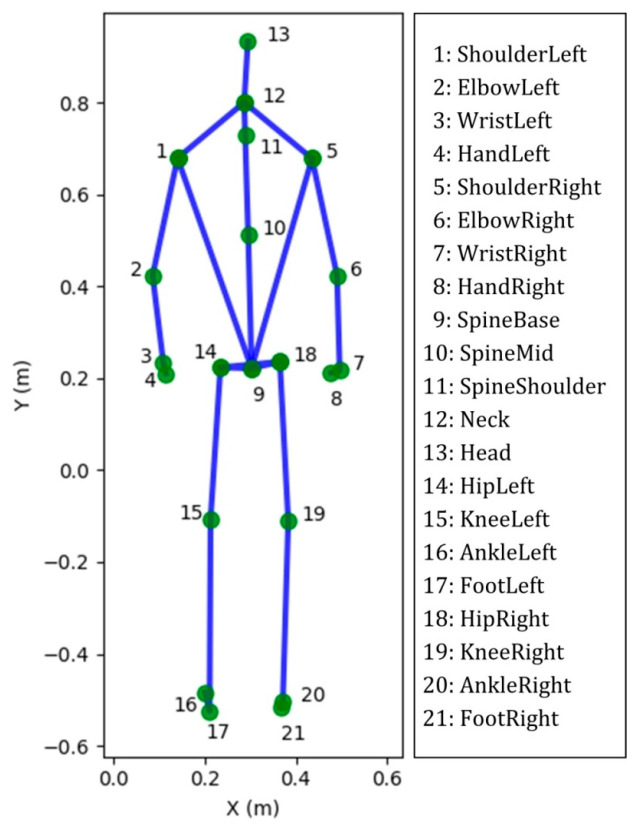
The 25 joints skeleton computed by Microsoft Kinect software. Twenty-one out of 25 joint labels, shown in standing position. Four very close joints are not illustrated, namely HandTipLeft, HandTipRight, ThumbLeft and ThumbRight.

**Figure 2:**
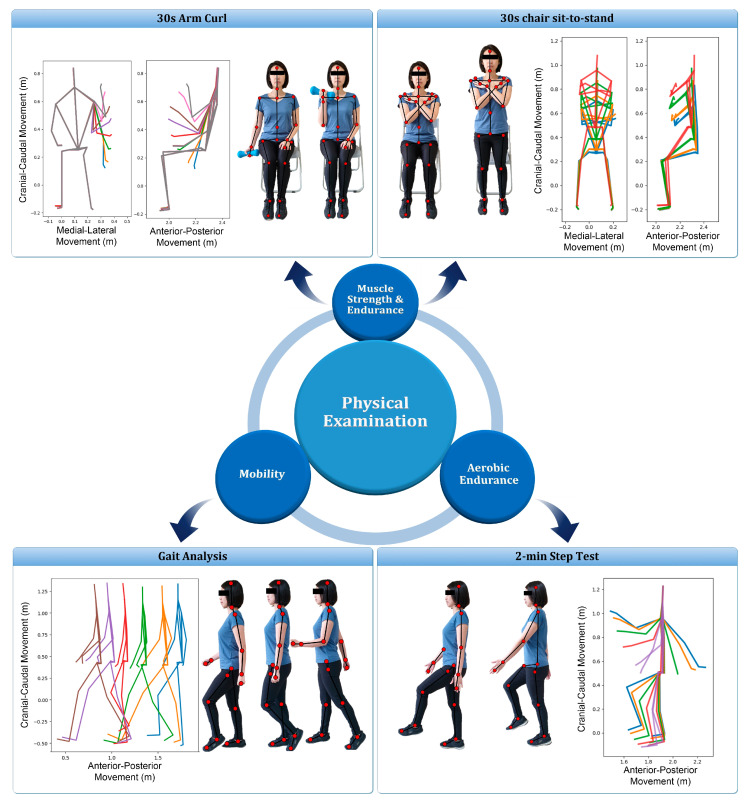
Illustration of the four functional assessment tests. For each test, multiple time frames of the skeleton pose were depicted from the acquired Kinect data of the present study.

The Kinect sensor (Microsoft Xbox One Kinect V2, Microsoft Corporation, Redmond, WA, USA) equipped with laboratory-developed software was used to capture the movement of joint centers (Figure 1) when performing the functional evaluation. Subjects performed four functional assessment tests, simultaneously monitored by a professional physical therapist and the Kinect system, including: (1) 30-s arm curl; (2) 30-s chair sit-to-stand (STS) test with arms folded across chest; (3) gait analysis at normal speed for 4 m; and (4) 2-min step test (Figure 2). These physical tests were selected to evaluate the muscular strength of upper body, lower body, ambulatory ability, and cardiopulmonary endurance, respectively [38,39,40,41]. To avoid the fatigue of the extremity, the sequence of the tests was performed from test No. 1 to 4 and two-minute rests among the tests were considered. Except gait analysis, which was repeated 3 times, the other test was performed once and all subjects could easily follow the guidance to perform the aforementioned experimental procedure (Figure 2).

### 2.2. Proposed Methodology

**Figure 3:**
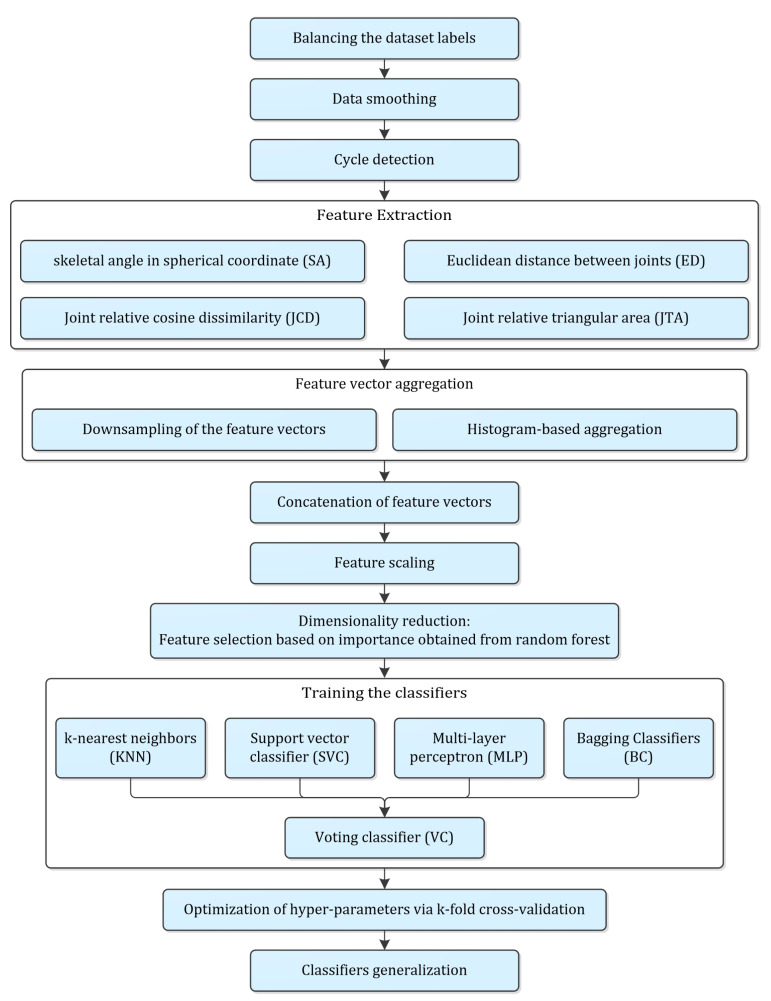
Flow diagram of the entire procedure proposed in the present study.

A detailed methodology was proposed to classify the frailty level based on the Kinect data, which is presented in Figure 3. It started with pre-processing stages including label balancing, data smoothing, cycle identification, feature extraction, time series aggregation, feature scaling and dimensionality reduction. Training of classifiers, hyper-parameter optimization and evaluation, and generalization of the models were the subsequent stages of the proposed methodology, which were explained accordingly.

#### 2.2.1. Balancing the Dataset Labels

Number of subjects with different frailty level (‘0′, ‘1′ or ‘2′) or gender classes (‘Male’ or ‘Female’) was quite diverse. This could lead to a bias in the classifier prediction toward the more frequent labels. In order to avoid such unrealistic bias, a random subject selector was applied to identify the same count of subjects for each of different classes. Additionally, our examination indicated that none of classifiers implemented in the present study were successful in accurate prediction of the frailty level ‘2′, which might refer to the small number of subjects with this label (20 subjects) compared to the number of individuals with the frailty levels ‘0′ and ‘1′ (323 and 444 subjects, respectively). Consequently, we focus on the levels ‘0′ and ‘1′ for the present study and postpone prediction of the level ‘2′ for future works by gathering more experimental data.

#### 2.2.2. Data Smoothing

**Figure 4:**
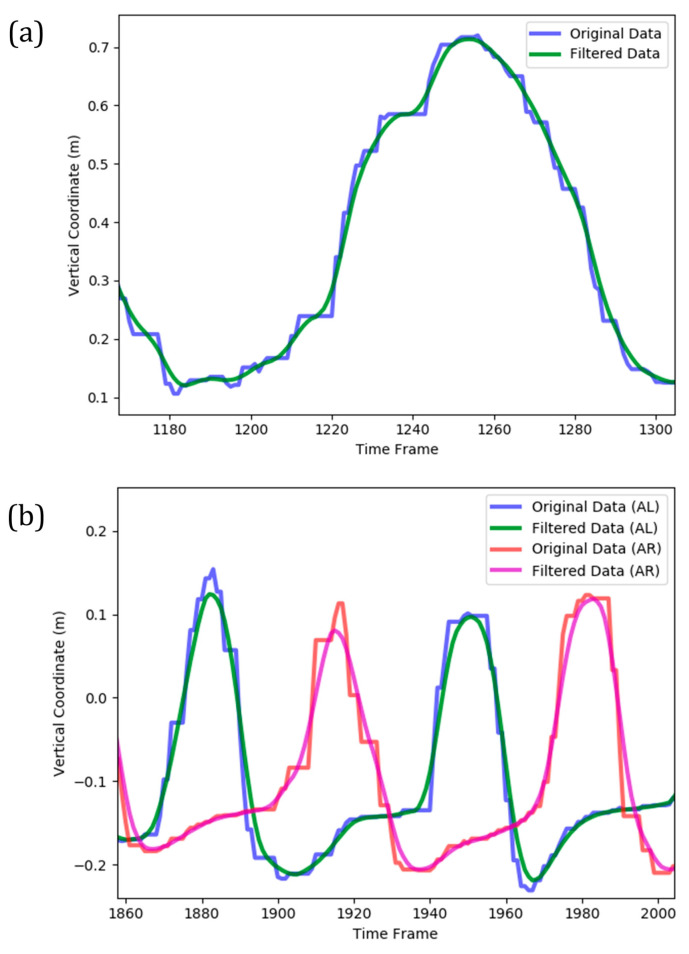
A sample for smoothing of Kinect data (vertical coordinate) by moving average Gaussian filter implemented on (**a**) a complete cycle of arm curl exercise; (**b**) two complete cycles of the two-minute steps exercise (AL and AR denote ‘AnkleLeft’ and ‘AnkleRight’ features, respectively).

Based on inherent sensors errors, the temporal evolution of joints’ 3D location was noisy and contained many local maximums and minimums. The presence of such fluctuations in the time series could complicate the machine learning model and make it more potential to overfitting. Additionally, the performance of cycle identification stage (as described in the following) deteriorates for a noisy time series. Consequently, it was necessary to smooth the data prior to further analysis. This was performed by applying a Gaussian moving average filter to the original Kinect data (Figure 4). The applied moving average filter reasonably smoothed the stepwise and fluctuative pattern of each time series, leading to a continuous trace of the overall trend. It also correctly followed the global extremums of temporal data, which was essential in the subsequent cycle identification stage.

#### 2.2.3. Cycle Detection

**Figure 5:**
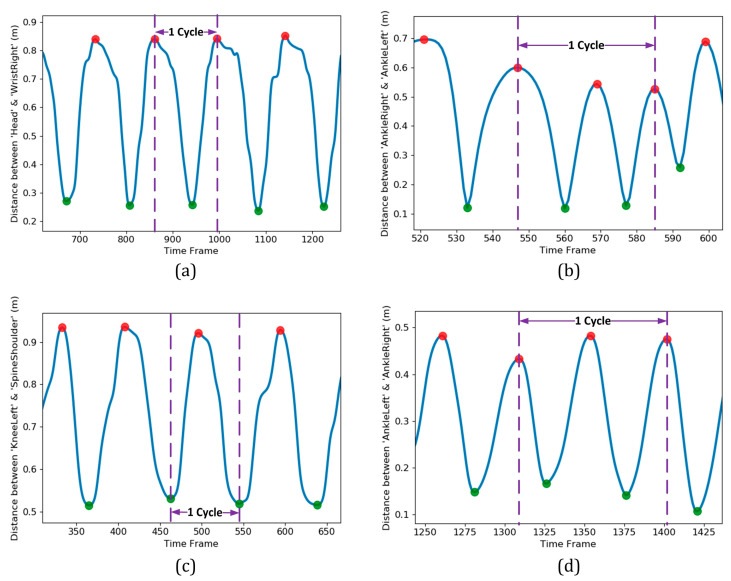
Temporal variation of distance metrics for the four different exercises of the present study: (**a**) ‘Arm Curled’; (**b**) ‘Gait Analysis’; (**c**) ‘Repeatedly Sit and Stand’; (**d**) ‘Two-minute Step’. For each of the exercises, one complete cycle was identified based on the extremum locations and the rules defined in Table 2.

Before extracting the features from the original data set, it was essential to identify and extract a portion of the time series that was consistent for various subjects. Accordingly, instead of using the entire time series for each exercise, it was quite crucial to identify one or a few cycles of it and do the subsequent process on that basis. For each exercise, a complete cycle was detected by monitoring the cyclic pattern of an appropriate distance metric. Figure 5 illustrates a few cycles of the four exercises in the present study. In each plot, the horizontal axis indicates the time frame index, while the vertical axis belongs to the distance metric utilized in cycle identification. The metrics and the portion of time series that could be considered as a complete cycle for each of the four exercises are summarized in Table 2.

The cycle identification approach introduced in Table 2 strictly depended on reliable detection of global maximums and minimums from each time series of the distance metric. Finding the global extremums from a pattern containing many local maximums and minimums was a challenging task. For this purpose, automatic multiscale-based peak detection (AMPD) as a robust algorithm was utilized [42]. The global extremums location found by the AMPD algorithm is illustrated in Figure 5 by circle markers (shown in red and green for maximums and minimums, respectively). The cycles were isolated based on the rules stated previously (Table 2) and a threshold reflected the significance of cyclic motion. The outcome for detection of one cycle for each of the four exercises is shown in Figure 5 by dashed lines as the left and right borders of the desirable portion of data.

#### 2.2.4. Feature Extraction

**Figure 6:**
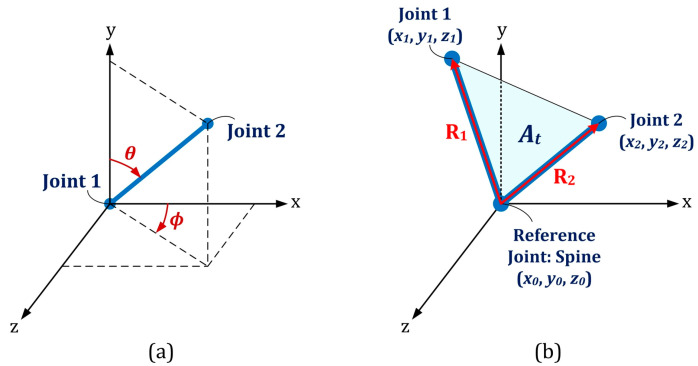
(**a**) Spherical coordinate representation; (**b**) Illustration of the three body joints used in the definition of cosine dissimilarity and joint triangular area.

The original Kinect data involves three-dimensional body joints coordinates that are not suitable for direct implementation as the input features of machine learning models. Regarding gait-based classifiers, various geometrical and kinematic features have been extracted in the literature. The proposed methodology in the present study was based on the following features extracted from the original acquired Kinect data:

1. **The skeletal angle in spherical coordinate** (**SA**): Two spherical coordinate angles (*ϕ* and *θ*) could be calculated for each pair of body joints with given Cartesian coordinates, namely $\left(x_{1},y_{1},z_{1}\right)$ and $\left(x_{2},y_{2},z_{2}\right)$, as shown in Figure 6a [28]:
  (2) $$\phi =\tan^{-1}\left(\frac{z_{2}-z_{1}}{x_{2}-x_{1}}\right)$$
  (3) $$\theta =\cos^{-1}\left(\frac{y_{2}-y_{1}}{l_{r}}\right)$$
  where $l_{r}$ is the Euclidean distance between the two joints,
  (4) $$l_{r}={\left[{\left(x_{2}-x_{1}\right)}^{2}+{\left(y_{2}-y_{1}\right)}^{2}+{\left(z_{2}-z_{1}\right)}^{2}\right]}^{1/2}$$
  Eight skeletal primitives, namely right- and left-side Humerus, Radius, Femur and Tibia were considered due to their dominant angular movements in different exercises. Based on this procedure, 16 feature vectors (8 vectors for each of angular components) were extracted, as well. They appeared as time-series with identical lengths as the original Kinect time series for each subject/exercise.
2. **Euclidean distance between joints** (**ED**): The distance between each pair of body joints can be calculated instantaneously using Equation (4) [30]. This feature was useful in both cycle identification procedure (as summarized in Table 2) and classification stage. The number of feature vectors extracted by this approach is $\left(N_{a}\right)\left(N_{a}-1\right)/2$ where $N_{a}$ was the number of active body joints, which was 21 by neglecting significance of the four body joints in the dynamics of the subject, namely left and right hand tip and thumb joints.
3. **Joint relative cosine dissimilarity** (**JCD**): This feature represented the cosine distance between two arbitrary body joints in three-dimensional vector space. It was capable to reflect the directional motion of every body joint with respect to the others [23]. $R_{1}$ and $R_{2}$ were three-dimensional vectors between a common reference point with coordinate $\left(x_{0},y_{0},z_{0}\right)$ and two arbitrary body joints with coordinates $\left(x_{1},y_{1},z_{1}\right)$ and $\left(x_{2},y_{2},z_{2}\right)$ as illustrated in Figure 6b. The cosine dissimilarity ($\delta_{c}$) was defined as:
  (5) $$\delta_{c}=1-\frac{R_{1}\cdot R_{2}}{\left\|R_{1}\right\|\left\|R_{2}\right\|}$$
  where ‖ ⋅ ‖ denotes the magnitude of each 3D vector. The spine body joint was considered as the reference point due to its stability in various skeletal movements. The cosine dissimilarity of each particular joint was calculated with respect to the remaining connected or non-connected body joints (except the spine joint, which is the reference point for all computations). The number of feature vectors obtained by this approach was $\left(N_{a}-1\right)\left(N_{a}-2\right)/2$.
4. **Joint relative triangular area** (**JTA**): Relative position of three body joints could be considered concurrently by definition of joint relative triangular area. It calculated the area of the triangle constructed by the spine joint (as the most stable body joint) and two other arbitrary joints (Figure 6b). Let $R_{1}$ and $R_{2}$ have the same definition as those described for the cosine dissimilarity, then the joint relative triangular area ($A_{t}$) was calculated as:
  (6) $$A_{t}=\frac{1}{2}\;\;\left\|R_{1}\times R_{2}\right\|$$
  Since this feature was based on the geometry area, it was more stable than distance- or angle-based features and could be more robust for a noisy data set [23]. Every three-joint combination (including the spine as the reference joint) based on 21 active body joints were considered and joint relative triangular area was calculated accordingly. This lead to $\left(N_{a}-1\right)\left(N_{a}-2\right)/2$ feature vectors for each subject/exercise.

#### 2.2.5. Feature Vector Aggregation and Concatenation

The Kinect data as multiple time series for different subjects/exercises had a variable length and included inconsistent frame rate for various recordings. The length of each time series was also quite large to be directly utilized as the feature vector. It was consequently mandatory to aggregate the time series extracted for each subject in order to downsample the data and convert it to a consistent feature vector with smaller and fixed length as the input of the classifier. Two types of aggregation procedure were implemented:

1. **Resampling the data to a lower frame rate** (**downsampling of the feature vectors**): After detection of a complete cycle, the segmented time series with different lengths for various subjects/exercises were resampled to a lower frame rate. Downsampling was performed by calculating the mean of data over each customized interval (bin) of the time series. Considering the number of bins to be $N_{B}$, a vector with the same length was obtained for each feature type.
2. **Histogram-based aggregation**: The histogram of each single- or multiple-cycle pattern in a predefined range was another way to downsample the original feature vector. The probability of data at each bin for a histogram with $N_{B}$ bins was then calculated to extract a vector with the same length as the aggregated vector for each feature type.
  After resampling each feature vector of a subject/exercise, all vectors should be concatenated into a unified feature vector for future stages. When all four feature types, namely SA, ED, JCD and JTA were implemented, the length of the concatenated feature vector was:
  (7) $$N_{f}=N_{B}\left(N_{SA}+N_{ED}+N_{JCD}+N_{JTA}\right)$$
  where $N_{SA}$, $N_{ED}$, $N_{JCD}$ and $N_{JTA}$ are the number of feature vectors regarding each of the four feature types, respectively.

#### 2.2.6. Scaling the Feature Space

Based on diversity of the scale of various feature types, it was crucial to implement some pre-processing on the input data to enhance consistency of various features scale and avoid any unfavorable effect of outliers in the training procedure. Standard normalization was implemented on each concatenated feature vector to transform it to a distribution with zero mean and unity variance.

#### 2.2.7. Curse of Dimensionality and Necessity of Dimensionality Reduction

Curse of dimensionality could be an important challenge in the Kinect-based classification, because performance of any classifier could be significantly deteriorated in high-dimensional feature space [43]. Existence of too many features with respect to small count of samples grows the risk of overfitting. Moreover, presence of many dimensions causes every sample to appear equidistant from the other ones, and adversely affects the classification quality. Accordingly, in order to enhance the classifier accuracy and avoid overfitting, it was crucial to systematically eliminate unimportant variables and select the dominant features. Principal component analysis (PCA) as a popular algorithm for dimensionality reduction could be a candidate for such purpose, but it cannot explicitly eliminate irrelevant features because of transformation of the variables into a new set of features ranked based on the extent of variance [44]. In contrast, random forest has emerged as a useful algorithm for feature selection by yielding feature importance measures for each variable. It is a practical approach for high dimensional data with a small number of samples [44,45,46,47]. The Gini importance metric (also known as feature importance) was utilized to measure the significance of each feature. This metric was directly obtained from the Gini index, a splitting function utilized in order to determine which attributes should be split during the learning stage. This factor quantifies the level of impurity/inequality of the samples assigned to each tree node based on the split done with respect to its parent. The features were sorted based on their Gini importance in a descending order in order to explicitly eliminate unimportant features and select a predefined number of features with higher significance ($N_{s}$) as the input variables for other classifiers, as successfully performed in numerous studies [45,46,48,49,50,51,52,53].

#### 2.2.8. Training the Classifiers

Frailty level classification based on machine learning approach has not been reported in the literature. We selected five classifiers based on advantages reported in the relevant literature regarding classification of Kinect time series data for various applications. They are summarized as follows:

1. **k-nearest neighbors** (**KNN**): The frailty class in this instance-based model was determined from the majority vote among all neighbors identified based on the distance metric calculated in the feature space [54]. Simplicity of implementation and low computational cost on KNN inspire using this classifier in cases with comparable accuracies to more complicated classifiers.
2. **Support vector classifier** (**SVC**): Using the so-called kernel trick approach to capture non-linear characteristics of the feature space, SVC provides a robust model that is suitable for classification of complex data sets with small to medium size [55]. This is particularly important for human skeleton data due to its inherent nonlinear characteristics that result in high-dimensional and multidisciplinary data [56]. SVC is robust to bias and variance of data, which are frequently observed in human gait or skeleton pose data, and results in accurate predictions for either binary or multiclass classifications [56]. The literature has also indicated that SVC is robust to overfitting and has a remarkable generalization capability [57].
3. **Multi-layer perceptron** (**MLP**): Implementation of numerous layers in conjunction with applying non-linear activation functions in MLP introduce it as a powerful tool in classification of non-linear problems. Flexibility of MLP in the learning process regardless of network structure, particularly in higher dimensions of feature space, and its reasonable classification accuracy are the other supportive reasons for selection of this classifier. Based on the literature in human gait patterns recognition, the MLP classifier has resulted in satisfactory predictions by comparable or even higher accuracies with respect to the SVC [58,59,60].
4. **Ensemble classifiers**: Aggregation of predictions performed by a group of classifiers could often enhance the accuracy compared to each individual classifier performance. Bagging and voting classifiers were two approaches among such ensemble learning methods, utilized in the present study.
  - **Bagging Classifier** (**BC**) **based on decision tree**: The bagging classifier works based on this principle that a group of classifiers can be combined to form a more reliable classifier [55]. Individual decision tree classifiers as the weak learners were trained on multiple random subsets of the dataset while bagging aggregates them to extract the majority vote of all the predictors as the output class and possibly enhance the classifier accuracy [55]. Implementation of bagging classifier based on decision tree on the Kinect data was reported to be successful in accurate recognition of physical disorder [61].
  - **Voting classifier** (**VC**): This ensemble approach aggregated the predictions made by multiple different classifiers (four previously mentioned classifiers) based on the predicted class probabilities to provide a more accurate classification. For instance, implementation of VC to predict freezing of gait in Parkinsonian patients using movement data from wearable sensors resulted in comparable accuracies to the SVM-based classifiers (as the most accurate estimators in that study) [62].

#### 2.2.9. Optimization of Hyper-Parameters via k-Fold Cross-Validation and Generalization

It was crucial to achieve the optimal values of the models hyper-parameters in a way to avoid overfitting and leads to suitable generalization performance for the model. In this regards, the random search approach was proved empirically and theoretically to be more efficient for hyper-parameter optimization than the grid search scheme [63] and consequently was applied in the present study. Accordingly, a wide range of hyper-parameters was considered, and a series of classifiers was trained based on randomly adopted hyper-parameters.

A k-fold cross validation procedure was employed to evaluate the model performance and in order to avoid occurrence of overfitting. After splitting the training data set (including N subjects) into k subsets of equal size, each subset with N/k samples was used for the model evaluation by feeding the remaining k−1 subsets as the input data to the model for training purpose. This was iterated until each of the k subsets had been exploited for validation. The average of the calculated errors for k validation subsets helped to stabilize the prediction accuracy estimated for the model and was used to determine the best hyper-parameters combination with the minimum error. It is noticeable that by increase of k as the number of folds, the difference in size between the training set and the resampled subsets got smaller, leading to reduction of the accuracy bias of the model. Simultaneously, this increase of k results in a higher variance of accuracy. There is no formal rule for selection of k, but it is conventionally set to an integer between 5 and 10 due to a trade-off between the bias and variance of the classifier accuracy.

After training the model and tuning the hyper-parameters, the model accuracy should be evaluated for the unseen data (subjects) in order to obtain the model performance in correct prediction of the frailty level (generalization of the model). The percentage of correct predictions made by the classifier compared to those obtained from the Fried’s frailty score (as the target labels) was considered as the model accuracy in either of the training, validation and generalization stages.

In order to validate the implemented methodology for frailty level classification, no existing results were available in the literature. Since we had no impression on the expectable accuracy for the present novel classification, in order to evaluate performance of our proposed methodology in comparison with the literature, we performed another classification to predict a different target label (the subject gender), but based on a similar input data to one of our exercises, namely the Kinect gait data. Although these two classification problems are not identical, their general structure is very similar. They both include extraction of relevant features from the original Kinect time series data, selection of appropriate attributes, feature scaling, training of classifiers, tuning of hyper-parameters and model generalizations. The main differences are the target labels and their correlations to the original Kinect data that could be handled in the training process of each classifier. The accuracy of classifiers in gender identification was evaluated based on two Kinect-based gait datasets:

1. The first was the data collected by Andersson and Araujo [29], known as Kinect gait biometry dataset, containing skeleton-based gait sequences of 140 subjects. Each walking time series has approximately 500–600 frames and 6–12 cycles.
2. The second one was the dataset of the present study, in which the gender of subjects was considered as the classification target class. The gait analysis data of at most 140 individuals out of 787 subjects was used in the gender identification procedure.

## 3. Results

### 3.1. Validation of Classifiers

Binary classification of the gait data based on 140 subjects of the mentioned datasets is presented in Table 3. The results obtained by applying our proposed methodology on the gait biometry dataset of Andersson and Araujo is compared to their classification results reported for three identical classifiers (KNN, SVC and MLP) [29]. Although two completely different methodologies were applied for feature extraction and pre-processing of data in our study and the work of Andersson and Araujo [29], close accuracies achieved by these two studies confirmed the reliability of our proposed approach in classification of the input Kinect data. It is also evident from Table 3 that the measured data of the present study has led to considerably better gender identification accuracy. Additionally, close accuracies are obtained by various classifiers for each configuration.

**Figure 7:**
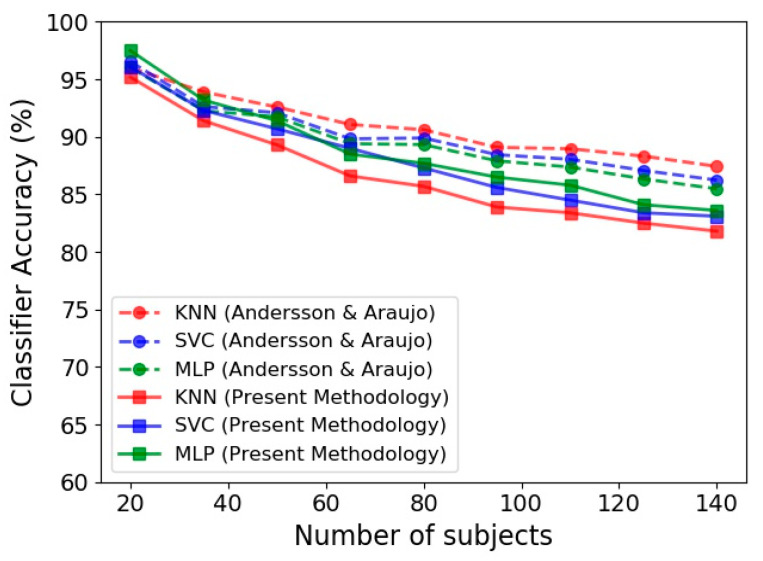
Accuracy of gender identification for three classifiers, namely KNN, SVC and MLP based on the Kinect gait biometry dataset, compared for our methodology (solid lines) with respect to that of Andersson and Araujo [29] (dashed lines).

Figure 7 presents the performance of KNN, SVC and MLP in terms of the number of subjects employed in the classification procedure. In this figure, the predictions achieved by the present methodology based on Kinect gait biometry dataset are compared to those reported by Andersson and Araujo [29]. An important observation in our results, that is in agreement with [29], is steady reduction of classification accuracy by increase of the number of subjects. This could be due to diversity of human skeletal dynamics that complicates the trained classification model. In such situation, using a complex model increases the training accuracy but at the same time increases the risk of overfitting. Tuning the hyper parameters and using appropriate training procedure to achieve a simpler model will circumvent the overfitting occurrence, but reduces the model accuracy (as is the case in Figure 7 for both our results and those of Andersson and Araujo [29]).

### 3.2. Frailty Level Classification Accuracy

**Figure 8:**
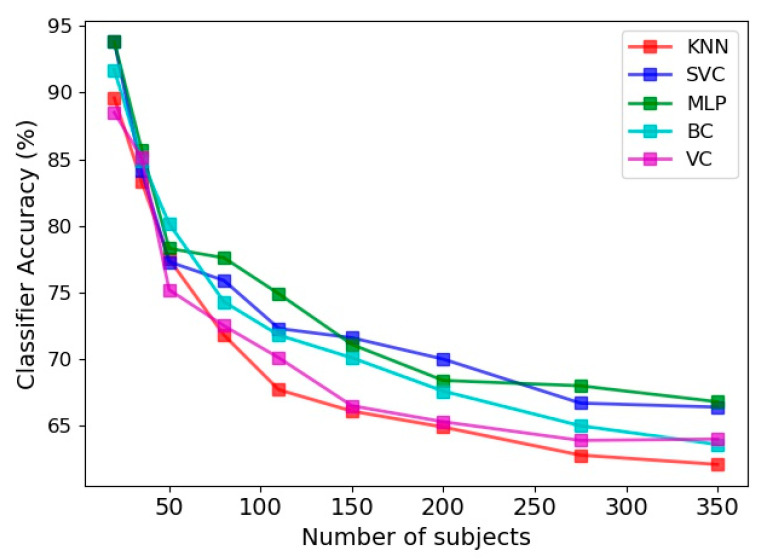
Accuracy of frailty level classification for five estimators, namely KNN, SVC, MLP, BC and VC for various number of subjects.

Utilizing four feature vectors, namely the limb angle, Euclidean distance between joints, joint relative cosine dissimilarity and joint relative triangular area, the accuracy of various classifiers in prediction of frailty level is presented in Figure 8. The 30 s sit-to-stand exercise was utilized and 100 features were selected as the input feature space. The accuracy was evaluated for various number of subjects utilized in the learning procedure, ranging from 20 to 350. Overall, the classification accuracy is smaller than the gender classification presented in the previous section.

Another point about Figure 8 is the steady reduction of classifiers accuracy with implementation of more numbers of subjects. This is in agreement with the findings on the gait biometry data of Andersson and Araujo [29], as discussed in the previous section. Comparison of different classifiers in Figure 8 also indicates that MLP and SVC have comparable accuracies, which are more successful than the remaining three classifiers in prediction of the frailty level. The least accuracy belongs to the KNN classifier, as was the case in gender classification.

**Figure 9:**
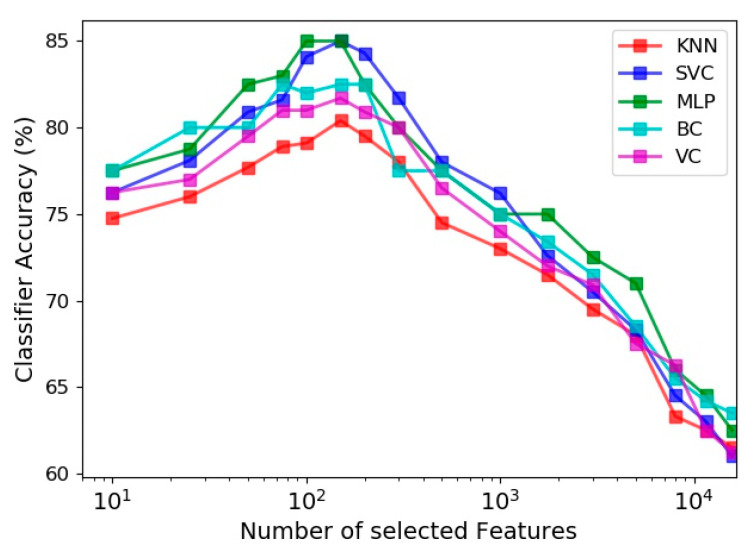
Effect of number of features selected by feature importance on the accuracy of different classifiers.

The effect of the number of features utilized in the learning process on the prediction accuracy of the five estimators is evaluated in Figure 9. Among all extracted feature vectors (including 15,700 vectors) from the 30 s sit-to-stand exercise data, different numbers of prominent vectors were identified and selected based on the Gini importance metric of the random forest classifier. Figure 9 indicates that the best accuracy of all classifiers happens by selecting about 150 most influential feature vectors out of 15,700 vectors, and exploiting more features deteriorates the model performance due to the curse of dimensionality. It is also noticeable for different estimators that using just 10 best feature vectors lead to accuracies, which are 5 to 8 percent less than their highest accuracies (obtained by using about 150 feature vectors).

**Figure 10:**
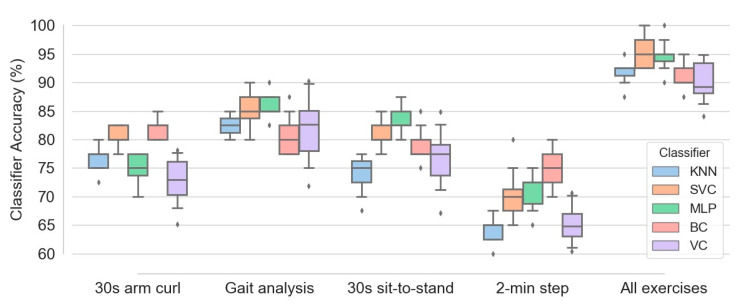
Box plots of various classifiers accuracy obtained based on five configurations of exercises, namely 30 s arm curl exercise, gait analysis, 30 s sit-to-stand exercise, 2 min step exercise and combination of all four exercise data.

Figure 10 illustrates the box plots of different classifiers’ accuracy calculated for the five configurations of exercises, namely 30 s arm curl exercise, gait analysis, 30 s sit-to-stand exercise, 2 min step exercise and combination of all four exercise data. One hundred subjects and all four feature types, namely SA, ED, JCD and JTA, were implemented in the learning procedure. Among four separate exercises, gait analysis causes best classification performance, with median accuracy in the range of 80 to 85 percent for various classifiers. This accuracy is slightly higher than those obtained by 30 s sit-to-stand and 30 s arm curl exercises (with comparable accuracies). In contrast, the 2 min step exercise data results the least accuracy (median accuracy in the range of 62.5 to 75 for different classifiers) in prediction of frailty level. It is also evident that aggregation of the entire data corresponding to all exercises causes a significant enhancement in the accuracy of all classifiers. This increases the median accuracy of SVC and MLP classifiers up to 95 percent. Additionally, the accuracy of various classifiers in the case of aggregated exercises is about 10 percent higher than those obtained from the gait analysis data (as the best single-exercise input data).

Regarding accuracy of different classifiers, Figure 10 shows that MLP, SVC and BC are among the most successful classifiers in the majority of configurations. In contrast, higher amount of interquartile range (the different between first and third quartiles) for most of box plots of the voting classifier indicates higher variance of classification accuracy of this estimator in comparison with the others.

**Figure 11:**
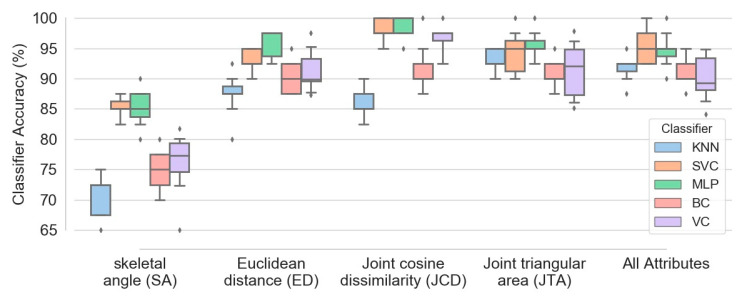
Box plots of different classifiers accuracy obtained based on five configurations of attributes, namely SA, ED, JCD, JTA and combination of all four attribute types.

Figure 11 demonstrates box plots of different classifiers accuracy obtained based on five configurations of features: (1) skeletal angle; (2) Euclidean distance; (3) joint cosine dissimilarity; (4) joint triangular area; (5) combination of all four feature types. The plots were extracted based on combination of all four exercises data and 100 subjects. Although aggregation of all mentioned attributes causes fairly accurate classification (with median accuracy higher than 89 percent for all five classifiers), Figure 11 confirms that utilizing more attribute types does not necessarily enhances the model performance. For instance, implementation of just JCD attribute is sufficient to obtain SVM and MLP classifiers with median accuracy of 97.5 percent, higher than any other configurations. The accuracy of bagging and voting classifiers (BC and VC) based on JCD attribute is also higher or at least comparable to other arrangements.

## 4. Discussion

A predictive model that can use simple physical tests would be beneficial for clinics to classify those individuals who are at risk of frailty in order to deliver the preventive interventions. The present study investigated the feasibility of the elderly frailty prediction based on the extracted data from functional assessment tests using the Kinect sensor. For this purpose, the ML methodology was utilized to provide a robust predictive model and different classification techniques were evaluated to achieve the most reliable and accurate ones. The strength of this study is the possibility to include the real-time skeletal movement data to develop the ML model, in contrast to the previous studies, which only used the administrative health database of clinical characteristics and socioeconomic factors [35,36]. To evaluate the accuracy of the model predictions, the Fried’s frailty score was used to identify the level of frailty for each individual which is the most well-known metric [64]. Although the Fried’s frailty phenotype is widely utilized to classify frailty in most of research investigations, some parameters of Fried’s frailty phenotype are based on self-reports (e.g., poor endurance) or can be interpreted differently by amateur individuals. On the other hand, the “weight loss” item in the definition of frailty score (Equation (1)) were not directly correlated with the skeletal movement which may be a limitation for pre-frail prediction. In this study, adequate accuracy for pre-frailty classification was achieved by the implemented ML modeling which confirms the applicability of this approach. Very simple assessment physical tests were selected for this study which can be performed without special utilities. Hence, it can be easily performed in small clinics or patients’ home as a remote assessment tool, which is the ultimate goal of this classification system.

The Kinect sensor was used in this study as a portable, low-cost, and practical instrument [19,65] to extract the skeletal movement data for elderly populations. The application of the Kinect sensor was previously validated to assess balance and impairment in the elderly people compared to the established standard tools such as marker based motion capture systems, force plates, and wearable sensors [64,66,67]. The accessibility of this device may make the proposed methodology in this study as a practical package which can be used both in clinics or remote telemedicine patient monitoring for frailty level identification. Simple and fast implementation of the ML modeling approach [23,26], and high accuracy obtained by above-mentioned classifications are positive characteristics of the present methodology. Hence, it can provide a promising solution which can be employed by users in clinics with no knowledge in programming or calculation of complex criteria. This encourages the development of a real-time automated classification package that receives 3D skeleton Kinect data and predicts the frailty level. This would be a reliable alternative to the classic methods working based on the traditional frailty scores.

Although prediction of the frailty level by ML classifiers was not performed in the literature, we utilized a well-known Kinect gait biometry dataset provided by Andersson and Araujo [29] in order to validate the performance of our proposed classifiers in gender prediction. Achievement of accuracies up to 84 percent by the proposed methodology of the present study, which was comparable by those reported by Andersson and Araujo [29], confirmed the reliability of our approach. Based on our acquired Kinect data in this study, the accuracy of classifiers in gender detection was even higher up to 92 percent which endorses the capability of the developed ML methodology.

Based on unavoidable errors in the tracking process, the raw skeleton data contains noise in registered joint positions [29,65]. Hence, it was observed that implementation of suitable pre-processing stages is essential for enhancement of the predictor accuracy. Removal of data noises and outliers, identification of consistent skeleton movement cycles for each participant and extraction of practical features from the raw data were major tasks that significantly enhanced the accuracy of our models. The outcome of these processes for each subject was input feature vectors that are consistent with those of other subjects in terms of fundamental characteristics. The feature space dimension was optimally selected via the Gini importance metric, because implementation of too many features in the learning procedure increases the risk of overfitting and would dramatically reduce the model accuracy. Finding the optimal hyper-parameters for each classifier through randomized search k-fold cross validation was also quite helpful regarding both accuracy enhancement and overfitting prevention. 

Among the four executed exercises in the present study, each one has a different contribution in the frailty level. It was found that gait analysis data caused the best accuracy, marginally better than the accuracy obtained from 30 s sit-to-stand and 30 s arm curl exercises. It is worthy to discuss that selected physical tests evaluates different abilities. The main purpose of the 30 s STS test relies on the strength and endurance of the lower extremity which is more relevant to the frailty expression [38,39,40,41]. However, the 2 min step test is more relevant to the aerobic ability of the elderly. It may be the reason for the observed variations for different performed physical tests. Furthermore, the results indicated that aggregation of the entire data corresponding to all four exercises was highly effective in enhancement of various classifiers accuracy (more than 10 percent of increase in classification accuracy). In contrast, implementation of a more extended variety of attribute types did not necessarily grow the frailty level identification accuracy. Utilization of joint cosine dissimilarity attribute solely resulted to 97.5 percent of median accuracy for the SVC and MLP as the best recognized predictors in the present study. The accuracy obtained by the voting classifier, either in terms of the median or variance, indicated that this could not be considered as a robust and accurate predictor of the frailty level. Regarding KNN, which was not generally an accurate classifier for frailty level, its simplicity of implementation and low computational cost encourage using of this classifier in cases with comparable accuracies, such as the case in which just JTA attribute is utilized as the feature space.

Some limitations of this study should be deliberated. The first limitation of the current study is frailty level imbalance of the subjects. Among 787 participants in our experiments, 323 subjects were recognized as ‘healthy’, 444 cases were assigned as ‘pre-frail’ and only 20 subjects were identified by ‘frail’ condition. Although a procedure was implemented to balance the dataset labels, existence of very few samples with the ‘frail’ label caused us to ignore the data corresponding to this label in the present study and postpone its prediction to our future research after gathering more data dealing with the ‘frail’ subjects. Another unavoidable limitation of this technique is due to this fact that the resolution of the captured data by the Kinect sensor decreases with distance [20,64]. Therefore, we designed the functional assessment tests protocol in an acceptable framework and re-checked the quality of the registered data before gathering the main experimental data. Third, the mini-mental state examination (MMSE) to assess the cognitive ability of the elderly was not performed in this study. However, all the tests were examined by a professional and well-trained physical therapist and all subjects could easily follow the guidance to perform the tests.

## 5. Conclusions

We developed a ML classifier model to identify the risk of frailty in elderly individuals based on evaluating the real-time skeletal movements using Kinect sensor. Our achieved results reveal that SVM and MLP classifiers can reliably and accurately distinguish the healthy subjects from the pre-frail ones which have potential to be recommended as consistent substitutions to the classic identification techniques. By applying the methodology used here, we can be hopeful that this technique can be used for remote patient monitoring and managing the rehabilitation exercises using virtual reality approaches. Further study is warranted to extend the clinical examinations of this methodology in larger populations.

## Figures and Tables

**Table 1:** Definition of frailty score and its characterizing parameters [2].

Parameter	Cardiovascular Health Study (CHS) Measure	Value/Criteria
Characterizing Parameters		
Weight Loss (*WL*)	Weight loss (unintentional loss greater than 10 lbs. in the past year)	0: No 1: Yes
Weakness (*WK*)	Grip strength: lowest 20% by gender and BMI (Based on the appendix of Fried et al. [2])	0: No 1: Yes
Poor Endurance (*PE*)	“Exhaustion” (self-report)	0: No 1: Yes
Slowness (*SL*)	Walking time speed/15 feet: slowest 20% by gender and height (Based on the appendix of Fried et al. [2])	0: No 1: Yes
Low Physical Activity (*LA*)	Kcals/week: lowest 20% Males: <383 Kcals/week Females: <270 Kcals/week	0: No 1: Yes
Frailty score definition and criteria		
Frailty score (*FS*)	Calculated based on the Fried definition (Equation (1))	0: healthy 1–2: Pre-frail 3–5: frail

**Table 2:** List of distance metrics used in cycle identification of various exercises.

Type of Exercise	Distance Metric Utilized to Identify the Cycle(s)	Portion of Time Series That Could Be Considered as a Complete Cycle	Additional Comments
Arm curled	3D distance between ‘Head’ and ‘WristRight’ or ‘WristLeft’, depending on the dominant hand	Starting from a global maximum and reaching the subsequent global maximum after passing a global minimum	The ‘Head’ position was utilized as fairly fixed reference coordinates that successfully help in detection of the cycle’s extremum points
Gait analysis	3D distance between left and right ankles coordinate	Starting from a global maximum and ending at its second subsequent global maximum	A maximum of distance metric corresponded to the farthest positions of the left and right ankles, while the minimum occurred when the ankles had the nearest positions
Repeatedly sit and stand	3D distance between ‘KneeLeft’ and ‘SpineShoulder’ coordinates	Starting from a global minimum and reaching the subsequent global minimum after passing a global maximum	The maximum of the distance metric corresponded to the standing position while the minimum occurred when the subject was sat on the bench
Two-minute step	3D distance between left and right ankles coordinate	Starting from a global maximum and ending at its second subsequent global maximum	Each maximum of distance metric corresponded to the highest vertical level of the lifting foot, while the minimum occurred in the standing position with both feet on the floor

**Table 3:** Accuracy of gender classification based on the Kinect gait biometry dataset of Andersson and Araujo [29] and the present dataset implementing 140 subjects.

Classifier	Dataset of Andersson and Araujo [29]		The Present Dataset
	Present Methodology	Andersson and Araujo Methodology [29]	
KNN	81.82	87.43	90.73
SVC	83.09	86.24	91.67
MLP	83.64	85.48	91.14
BAG	82.62	NA	92.03
VC	83.22	NA	90.85

## Data Availability

The data of the present work is available on demand from the corresponding author.

## References

[1] Xue Q.-L. (2011). The frailty syndrome: Definition and natural history. Clin. Geriatr. Med..

[2] Fried L.P., Tangen C.M., Walston J., Newman A.B., Hirsch C., Gottdiener J., Seeman T., Tracy R., Kop W.J., Burke G. (2001). Frailty in Older Adults: Evidence for a Phenotype. J. Gerontol. Ser. A Biol. Sci. Med. Sci..

[3] Liu C.K., Fielding R.A. (2011). Exercise as an Intervention for Frailty. Clin. Geriatr. Med..

[4] Parvaneh S., Mohler J., Toosizadeh N., Grewal G.S., Najafi B. (2017). Postural Transitions during Activities of Daily Living Could Identify Frailty Status: Application of Wearable Technology to Identify Frailty during Unsupervised Condition. Gerontology.

[5] Fugate Woods N., LaCroix A.Z., Gray S.L., Aragaki A., Cochrane B.B., Brunner R.L., Masaki K., Murray A., Newman A.B. (2005). Frailty: Emergence and Consequences in Women Aged 65 and Older in the Women’s Health Initiative Observational Study. J. Am. Geriatr. Soc..

[6] Bouillon K., Kivimaki M., Hamer M., Sabia S., Fransson E.I., Singh-Manoux A., Gale C.R., Batty G.D. (2013). Measures of frailty in population-based studies: An overview. BMC Geriatr..

[7] Ibrahim K., Howson F.F.A., Culliford D.J., Sayer A.A., Roberts H.C. (2019). The feasibility of assessing frailty and sarcopenia in hospitalised older people: A comparison of commonly used tools. BMC Geriatr..

[8] Rockwood K. (2005). A global clinical measure of fitness and frailty in elderly people. Can. Med. Assoc. J..

[9] Rothman M.D., Leo-Summers L., Gill T.M. (2008). Prognostic Significance of Potential Frailty Criteria. J. Am. Geriatr. Soc..

[10] Pannurat N., Thiemjarus S., Nantajeewarawat E. (2014). Automatic Fall Monitoring: A Review. Sensors.

[11] Ezeugwu V., Klaren R.E., Hubbard E.A., Manns P., Motl R.W. (2015). Mobility disability and the pattern of accelerometer-derived sedentary and physical activity behaviors in people with multiple sclerosis. Prev. Med. Rep..

[12] Elmannai W., Elleithy K. (2017). Sensor-Based Assistive Devices for Visually-Impaired People: Current Status, Challenges, and Future Directions. Sensors.

[13] Almeida O., Zhang M., Liu J.-C. Dynamic Fall Detection and Pace Measurement in Walking Sticks. Proceedings of the Joint Workshop on High Confidence Medical Devices, Software, and Systems and Medical Device Plug-and-Play Interoperability.

[14] Nikkhoo M., Niu C.-C., Fu C.-J., Lu M.-L., Chen W.-C., Lin Y.-H., Cheng C.-H. (2020). Reliability and Validity of a Mobile Device for Assessing Head Control Ability. J. Med. Biol. Eng..

[15] Delahoz Y., Labrador M. (2014). Survey on Fall Detection and Fall Prevention Using Wearable and External Sensors. Sensors.

[16] Aurand A.M., Dufour J.S., Marras W.S. (2017). Accuracy map of an optical motion capture system with 42 or 21 cameras in a large measurement volume. J. Biomech..

[17] Colyer S.L., Evans M., Cosker D.P., Salo A.I.T. (2018). A Review of the Evolution of Vision-Based Motion Analysis and the Integration of Advanced Computer Vision Methods Towards Developing a Markerless System. Sports Med. Open.

[18] Mundher Z.A., Jiaofei Z. (2014). A Real-Time Fall Detection System in Elderly Care Using Mobile Robot and Kinect Sensor. Int. J. Mater. Mech. Manuf..

[19] Guerra B.M.V., Ramat S., Gandolfi R., Beltrami G., Schmid M. Skeleton data pre-processing for human pose recognition using Neural Network. Proceedings of the 2020 42nd Annual International Conference of the IEEE Engineering in Medicine & Biology Society (EMBC).

[20] Choppin S., Wheat J. (2013). The potential of the Microsoft Kinect in sports analysis and biomechanics. Sports Technol..

[21] Stamm O., Heimann-Steinert A. (2020). Accuracy of Monocular Two-Dimensional Pose Estimation Compared With a Reference Standard for Kinematic Multiview Analysis: Validation Study. JMIR Mhealth Uhealth.

[22] Baeza-Barragán M.R., Labajos Manzanares M.T., Ruiz Vergara C., Casuso-Holgado M.J., Martín-Valero R. (2020). The Use of Virtual Reality Technologies in the Treatment of Duchenne Muscular Dystrophy: Systematic Review. JMIR Mhealth Uhealth.

[23] Bari A.S.M.H., Gavrilova M.L. (2019). Artificial Neural Network Based Gait Recognition Using Kinect Sensor. IEEE Access.

[24] Halilaj E., Rajagopal A., Fiterau M., Hicks J.L., Hastie T.J., Delp S.L. (2018). Machine learning in human movement biomechanics: Best practices, common pitfalls, and new opportunities. J. Biomech..

[25] Kidziński Ł., Yang B., Hicks J.L., Rajagopal A., Delp S.L., Schwartz M.H. (2020). Deep neural networks enable quantitative movement analysis using single-camera videos. Nat. Commun..

[26] Kastaniotis D., Theodorakopoulos I., Economou G., Fotopoulos S. (2016). Gait based recognition via fusing information from Euclidean and Riemannian manifolds. Pattern Recognit. Lett..

[27] Kastaniotis D., Theodorakopoulos I., Theoharatos C., Economou G., Fotopoulos S. (2015). A framework for gait-based recognition using Kinect. Pattern Recognit. Lett..

[28] Kastaniotis D., Theodorakopoulos I., Economou G., Fotopoulos S. Gait-based gender recognition using pose information for real time applications. Proceedings of the 2013 18th International Conference on Digital Signal Processing (DSP).

[29] Andersson V., Araujo R. Person identification using anthropometric and gait data from kinect sensor. Proceedings of the AAAI Conference on Artificial Intelligence.

[30] Rahman M.W., Gavrilova M.L. Kinect gait skeletal joint feature-based person identification. Proceedings of the 2017 IEEE 16th International Conference on Cognitive Informatics & Cognitive Computing (ICCI* CC).

[31] Chen K., Vervoort D., Vuillerme N., Kosse N., Hortobágyi T., Lamoth C.J.C. (2016). Multivariate Analyses and Classification of Inertial Sensor Data to Identify Aging Effects on the Timed-Up-and-Go Test. PLoS ONE.

[32] Greene B.R., Doheny E.P., O’Halloran A., Anne Kenny R. (2013). Frailty status can be accurately assessed using inertial sensors and the TUG test. Age Ageing.

[33] Collado-Villaverde A., R-Moreno M.D., Barrero D.F., Rodriguez D. (2017). Machine Learning Approach to Detect Falls on Elderly People Using Sound. Advances in Artificial Intelligence: From Theory to Practice.

[34] Lovis C., Albert M.V., Kording K., Herrmann M., Jayaraman A. (2012). Fall Classification by Machine Learning Using Mobile Phones. PLoS ONE.

[35] Tarekegn A., Ricceri F., Costa G., Ferracin E., Giacobini M. (2020). Predictive Modeling for Frailty Conditions in Elderly People: Machine Learning Approaches. JMIR Med. Inform..

[36] Ambagtsheer R.C., Shafiabady N., Dent E., Seiboth C., Beilby J. (2020). The application of artificial intelligence (AI) techniques to identify frailty within a residential aged care administrative data set. Int. J. Med. Inform..

[37] Peng L.-N., Hsiao F.-Y., Lee W.-J., Huang S.-T., Chen L.-K. (2020). Comparisons Between Hypothesis- and Data-Driven Approaches for Multimorbidity Frailty Index: A Machine Learning Approach. J. Med. Internet Res..

[38] Rikli R.E., Jones C.J. (1999). Development and Validation of a Functional Fitness Test for Community-Residing Older Adults. J. Aging Phys. Act..

[39] Schwenk M., Mohler J., Wendel C., D’Huyvetter K., Fain M., Taylor-Piliae R., Najafi B. (2015). Wearable Sensor-Based In-Home Assessment of Gait, Balance, and Physical Activity for Discrimination of Frailty Status: Baseline Results of the Arizona Frailty Cohort Study. Gerontology.

[40] Alcazar J., Losa-Reyna J., Rodriguez-Lopez C., Alfaro-Acha A., Rodriguez-Mañas L., Ara I., García-García F.J., Alegre L.M. (2018). The sit-to-stand muscle power test: An easy, inexpensive and portable procedure to assess muscle power in older people. Exp. Gerontol..

[41] Baltasar-Fernandez I., Alcazar J., Rodriguez-Lopez C., Losa-Reyna J., Alonso-Seco M., Ara I., Alegre L.M. (2021). Sit-to-stand muscle power test: Comparison between estimated and force plate-derived mechanical power and their association with physical function in older adults. Exp. Gerontol..

[42] Scholkmann F., Boss J., Wolf M. (2012). An efficient algorithm for automatic peak detection in noisy periodic and quasi-periodic signals. Algorithms.

[43] Wu Y., Zhang A. Feature selection for classifying high-dimensional numerical data. Proceedings of the 2004 IEEE Computer Society Conference on Computer Vision and Pattern Recognition.

[44] Menze B.H., Kelm B.M., Masuch R., Himmelreich U., Bachert P., Petrich W., Hamprecht F.A. (2009). A comparison of random forest and its Gini importance with standard chemometric methods for the feature selection and classification of spectral data. BMC Bioinform..

[45] Chen R.-C., Dewi C., Huang S.-W., Caraka R.E. (2020). Selecting critical features for data classification based on machine learning methods. J. Big Data.

[46] Kawakubo H., Yoshida H. (2012). Rapid feature selection based on random forests for high-dimensional data. Expert Syst. Appl..

[47] Archer K.J., Kimes R.V. (2008). Empirical characterization of random forest variable importance measures. Comput. Stat. Data Anal..

[48] Saeys Y., Inza I., Larranaga P. (2007). A review of feature selection techniques in bioinformatics. Bioinformatics.

[49] Dindorf C., Konradi J., Wolf C., Taetz B., Bleser G., Huthwelker J., Drees P., Fröhlich M., Betz U. (2020). General method for automated feature extraction and selection and its application for gender classification and biomechanical knowledge discovery of sex differences in spinal posture during stance and gait. Comput. Methods Biomech. Biomed. Eng..

[50] Ebina T., Toh H., Kuroda Y. (2011). DROP: An SVM domain linker predictor trained with optimal features selected by random forest. Bioinformatics.

[51] Chang Y., Wang Y., Chen C., Ricanek K. (2011). Improved image-based automatic gender classification by feature selection. J. Artif. Intell. Soft Comput. Res..

[52] Chen Y.-W., Lin C.-J. (2006). Combining SVMs with various feature selection strategies. Feature Extraction.

[53] Rahman M.S., Rahman M.K., Kaykobad M., Rahman M.S. (2018). isGPT: An optimized model to identify sub-Golgi protein types using SVM and Random Forest based feature selection. Artif. Intell. Med..

[54] Mohammed M., Khan M.B., Bashier E.B.M. (2016). Machine Learning: Algorithms and Applications.

[55] Marsland S. (2015). Machine Learning: An Algorithmic Perspective.

[56] Figueiredo J., Santos C.P., Moreno J.C. (2018). Automatic recognition of gait patterns in human motor disorders using machine learning: A review. Med. Eng. Phys..

[57] Khera P., Kumar N. (2020). Role of machine learning in gait analysis: A review. J. Med. Eng. Technol..

[58] Zhang Y., Ma Y. (2019). Application of supervised machine learning algorithms in the classification of sagittal gait patterns of cerebral palsy children with spastic diplegia. Comput. Biol. Med..

[59] Chan H., Yang M., Wang H., Zheng H., McClean S., Sterritt R., Mayagoitia R.E. (2013). Assessing gait patterns of healthy adults climbing stairs employing machine learning techniques. Int. J. Intell. Syst..

[60] Pogorelc B., Bosnić Z., Gams M. (2012). Automatic recognition of gait-related health problems in the elderly using machine learning. Multimed. Tools Appl..

[61] Saha S., Pal M., Konar A., Roy J. (2015). Ensemble Classifier-Based Physical Disorder Recognition System Using Kinect Sensor. Computational Advancement in Communication Circuits and Systems.

[62] Arami A., Poulakakis-Daktylidis A., Tai Y.F., Burdet E. (2019). Prediction of gait freezing in Parkinsonian patients: A binary classification augmented with time series prediction. IEEE Trans. Neural. Syst. Rehabil. Eng..

[63] Bergstra J., Bengio Y. (2012). Random search for hyper-parameter optimization. J. Mach. Learn. Res..

[64] Panhwar Y.N., Naghdy F., Naghdy G., Stirling D., Potter J. (2019). Assessment of frailty: A survey of quantitative and clinical methods. BMC Biomed. Eng..

[65] Barreira C.C., Forner-Cordero A., Grangeiro P.M., Moura R.T. (2020). Kinect v2 based system for gait assessment of children with cerebral palsy in rehabilitation settings. J. Med. Eng. Technol..

[66] Yeung L.F., Cheng K.C., Fong C.H., Lee W.C.C., Tong K.-Y. (2014). Evaluation of the Microsoft Kinect as a clinical assessment tool of body sway. Gait Posture.

[67] Eltoukhy M.A., Kuenze C., Oh J., Signorile J.F. (2018). Validation of Static and Dynamic Balance Assessment Using Microsoft Kinect for Young and Elderly Populations. IEEE J. Biomed. Health Inform..

